# Targeting mitochondrial homeostasis in the treatment of non-alcoholic fatty liver disease: a review

**DOI:** 10.3389/fphar.2024.1463187

**Published:** 2024-09-03

**Authors:** Yalan Deng, Yuan Dong, Sitian Zhang, Yingmei Feng

**Affiliations:** ^1^ Department of Science and Technology, Beijing Youan Hospital, Capital Medical University, Beijing, China; ^2^ Laboratory for Clinical Medicine, Capital Medical University, Beijing, China; ^3^ School of Basic Medical Sciences, Capital Medical University, Beijing, China

**Keywords:** mitochondrial homeostasis, non-alcoholic fatty liver disease (NAFLD), non-alcoholic steatohepatitis (NASH), drug target, hepatocytes

## Abstract

Non-alcoholic fatty liver disease (NAFLD) is the most common chronic liver disease worldwide, and its prevalence is rapidly increasing. Antioxidants, lipid-lowering medications, and lifestyle interventions are the most commonly used treatment options for NAFLD, but their efficacy in inhibiting steatosis progression is limited and their long-term ineffectiveness and adverse effects have been widely reported. Therefore, it is important to gain a deeper understanding of the pathogenesis of NAFLD and to identify more effective therapeutic approaches. Mitochondrial homeostasis governs cellular redox biology, lipid metabolism, and cell death, all of which are crucial to control hepatic function. Recent findings have indicated that disruption of mitochondrial homeostasis occurs in the early stage of NAFLD and mitochondrial dysfunction reinforces disease progression. In this review, we summarize the physical roles of the mitochondria and describe their response and dysfunction in the context of NAFLD. We also discuss the drug targets associated with the mitochondria that are currently in the clinical trial phase of exploration. From our findings, we hope that the mitochondria may be a promising therapeutic target for the treatment of NAFLD.

## Introduction

Non-alcoholic fatty liver disease (NAFLD) is a chronic liver disease, featured with lipid accumulation in the liver and hepatic steatosis based on imaging or histology, while excluding secondary causes of hepatic steatosis such as significant alcohol consumption (Rong et al., 2022). In 2020, a broader term, metabolic (dysfunction)-associated fatty liver disease (MAFLD), was suggested to replace NAFLD, with expanded diagnostic criteria. This updated terminology better emphasizes the significance of metabolic dysfunction in the disease’s development (Shiha et al., 2021). In terms of disease progression, the stages of NAFLD comprise non-alcoholic fatty liver (NAFL), non-alcoholic steatohepatitis (NASH), liver fibrosis, and cirrhosis (Loomba et al., 2021). It is estimated that the prevalence of NAFLD has reached 25% among the global population (Younossi et al., 2019). Therefore, NAFLD is a global public health concern. Hepatocytes are metabolically active cells with enriched mitochondria. Mitochondrial homeostasis governs cell function through various mechanisms, including cellular division, oxidative stress, autophagy, and mitochondrial quality control (Kumar et al., 2021). In this review, we summarize the physical properties of the mitochondria and describe how disrupted mitochondrial homeostasis participates in the pathogenesis of NAFLD.

## Mitochondrial structure and function in hepatocytes

The mitochondria are two-layered membrane-coated organelles present in most eukaryotic cells. They possess their own genetic material, and they are a semi-autonomous organelle with a limited genome size. In general, they are approximately 0.5–1.0 μm in diameter, although this varies among species. Nevertheless, the structure of the mitochondria is almost identical among species, comprising the outer mitochondrial membrane, mitochondrial membrane space, inner mitochondrial membrane, and mitochondrial matrix. The outer mitochondrial membrane (OMM) is smooth and acts as the organelle boundary. The inner mitochondrial membrane (IMM) folds inward to form a mitochondrial crest, which participates in biochemical reactions. The mitochondrial matrix is located in the space between the outer and inner membranes (Clare et al., 2022).

The mitochondria are considered as the “powerhouses” of cells. The chemical reactions of the tricarboxylic acid (TCA) cycle, oxidative phosphorylation (OXPHOS), and fatty acid oxidation occur in the mitochondria. Adenosine triphosphate (ATP) is generated by utilizing the electrochemical gradient across the inner mitochondrial membrane, which is produced by the electron transport chain (ETC). Beyond energy production, hepatocytes are actively involved in glucose metabolism (Wu et al., 2019), lipid metabolism (Dong et al., 2024), cholesterol synthesis (Goicoechea et al., 2023), and detoxification and excretion (da Silva Lima et al., 2022; Ramanathan et al., 2022; Chen et al., 2024). In addition, the mitochondria are involved in multiple biological processes, such as cell death and differentiation. To be noted, reactive oxygen species (ROS) are mainly produced in the mitochondria, and they contribute to different forms of cell death. For instance, downstream of apoptotic signals, the mitochondrial outer membrane becomes permeabilized and cytochrome-c is released into the cytosol for subsequent caspase activation (Bock and Tait, 2020). Furthermore, excessive accumulation of lipid peroxides on the cell membrane disturbs iron metabolism and stimulates ROS production. Hydrogen peroxide (O2•−) reacts with ferrous ions to accelerate polyunsaturated fatty acid, hydroperoxide formation in the mitochondria for ferroptosis (Gan, 2021).

**FIGURE 1:**
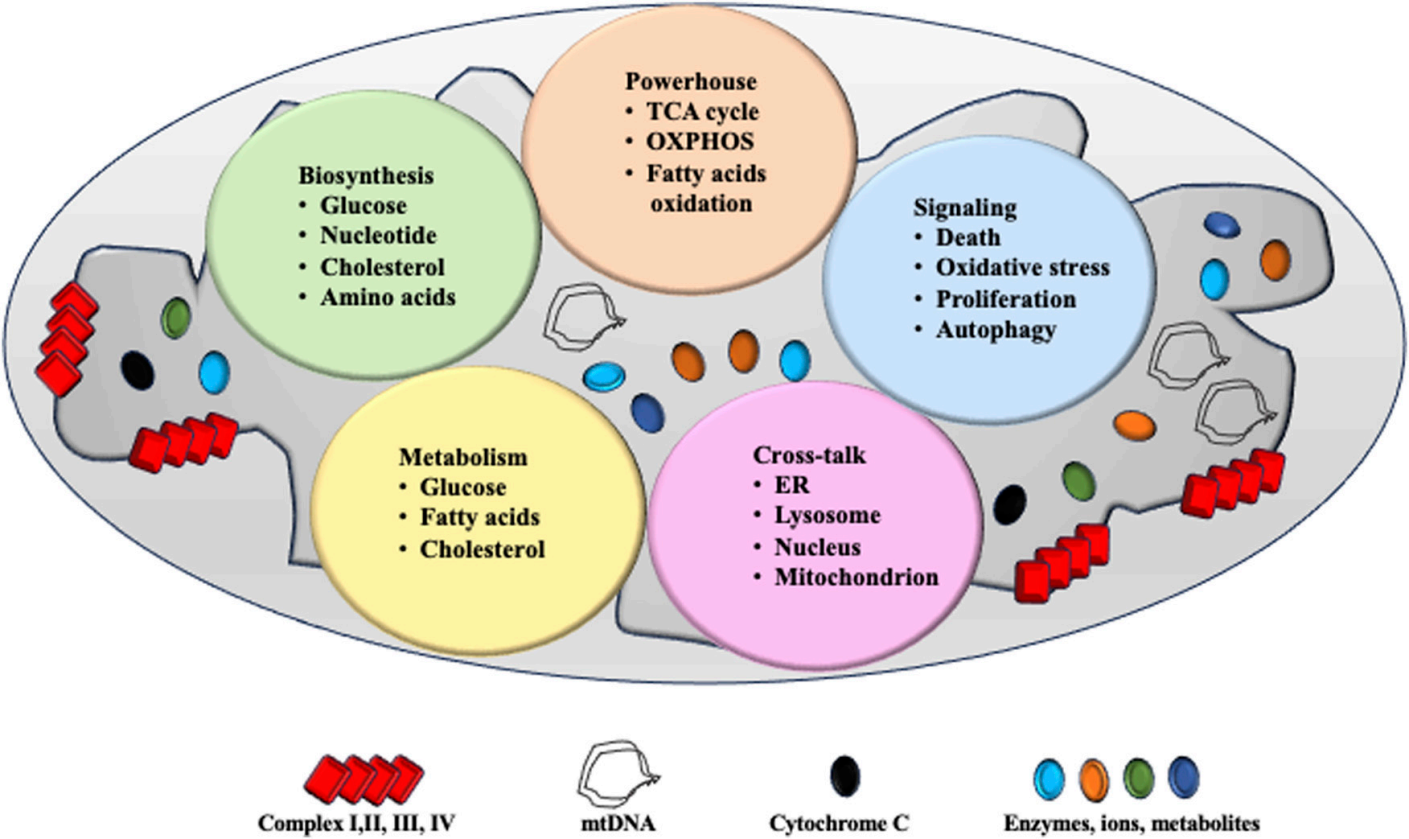
Mitochondrial structure and function. The mitochondria are two-layered membrane-coated organelles present in most eukaryotic cells. Comprising the outer mitochondrial membrane, mitochondrial membrane space, inner mitochondrial membrane, and mitochondrial matrix. Mitochondria serve as powerhouse for cell function and ATP is produced via tricarboxylic acid (TCA) cycle, OXPHOS system and fatty acid oxidation. Mitochondria are a critical site for cell biosynthesis, metabolism and cell-signaling. Communications between mitochondria and other organelles help maintain their homeostasis and function.

Mitochondria structure and function is summarized in Figure 1.

### Hepatic mitochondrial homeostasis

Mitochondrial homeostasis is delicately controlled by a set of intrinsic mechanisms that safeguard mitochondrial integrity and function. This system includes processes such as biogenesis, mitochondrial fission and fusion, mitophagy, and redox regulation (Li et al., 2020).

#### Hepatic mitochondrial biogenesis

The mitochondrial genome is known as mitochondrial double-stranded circular DNA (mtDNA). mtDNA encodes 13 proteins involved in the ETC, all of which are subunits of enzyme complexes involved in OXPHOS (Kremer and Rehling, 2024). In addition, nuclear genes encode the mammalian mitochondrial proteome, which consists of approximately 1,000–1,500 distinct proteins (Pfanner et al., 2019). Once released into the cytosol, they target the submitochondrial compartment to assemble mitochondrion (Pfanner et al., 2019).

Permanent mtDNA replication is a crucial step in mitochondrial biogenesis, which is activated by the peroxisome proliferator-activated receptor-γ co-activator-1 proteins (PGC-1α and PGC-1β). Among the PGC-1 family members, PGC-1α is the most important one. Following PGC-1α activation, transcriptional factors, including nuclear respiratory factors 1 and 2, estrogen-related receptor-α, and mitochondrial transcription factor 1 (TFAM), are activated (Rubio-Cosials et al., 2011; Ploumi et al., 2017). TFAM, together with its interacting proteins in the initiation complex, binds to specific promoters and induces structural changes in DNA strands for RNA polymerase recognition, resulting in mtDNA replication and transcription (Rubio-Cosials et al., 2011).

Adenosine monophosphate-activated protein kinase (AMPK) and Sirt-1 regulate PGC-1α activity by posttranslational phosphorylation and deacetylation, respectively (Ploumi et al., 2017; Zeng and Chen, 2022). A previous study showed that in cultured neonatal rat ventricular cardiomyocytes, treatment of C1q/tumor necrosis factor-related protein-3 promoted mitochondrial biogenesis via the AMPK/PGC-1α pathway (Zhang et al., 2017). In a murine model of partial hepatic ischemia and reperfusion, the administration of the anti-oxidant agent nobiletin protected hepatocyte damage by enhancing the PGC-1α pathway, which was abolished by Sirt-1 inhibition (Dusabimana et al., 2019).

#### Hepatic mitochondrial dynamics

The mitochondria undergo continuous fission and fusion, which is referred to as mitochondrial dynamics. Mitochondrial fission is defined as the division of one mitochondrion into two mitochondria, whereas mitochondrial fusion denotes the formation of one mitochondrion from two mitochondria. Fission and fusion events can be influenced by metabolic conditions and are regulated by different proteins.

When exposed to nutrient-rich environmental conditions, hepatocytes tend to keep their mitochondria in a separated (fragmented) state to prevent energy waste, reducing bioenergetic efficiency and augmenting mitochondrial uncoupling, all of which leads to a simultaneous increase in nutrient storage (Adebayo et al., 2021). Mitochondrial fission is facilitated by mitochondrial fission factor (Takeichi et al., 2021), fission protein 1, and dynamin-related protein 1 (Du et al., 2017; Stevanović et al., 2020).

On the contrary, under conditions of nutrient deprivation, the hepatic mitochondria remain in the connected (elongated) state for a longer duration (Adebayo et al., 2021). Mitochondrial fusion is considered a protective mechanism against metabolic and environmental stress in which gene products are transferred between the mitochondria to maintain optimal function. Mitochondrial fusion in hepatocytes is controlled by mitofusin proteins (Mfn-1 and Mfn-2) (Zhang et al., 2010; Gong et al., 2019).

#### Hepatic mitophagy

Mitochondrial biogenesis and mitophagy work together to maintain mitochondrial homeostasis. As a specialized type of autophagy, mitochondrial mitophagy controls mitochondrial turnover and recycling, ensuring hepatic homeostasis by eliminating long-lived or damaged mitochondria (Xian and Liou, 2021). Three primary mitophagy pathways have been identified: PTEN-induced kinase (PINK1) and ubiquitin E3 ligase Parkin, Bcl-2 interacting protein 3 (BNIP3)/light chain 3B (LC3B), and FUN14 domain-containing 1 (FUNDC1) (Springer et al., 2021; Agarwal and Muqit, 2022; Bi et al., 2024). In response to mitochondria membrane potential reduction, PINK1 is accumulated in OMM that recruits Parkin translocation from cytosol to mitochondria for protein degradation (Agarwal and Muqit, 2022). BNIP3 protein is required for glucagon/fasting-triggered mitophagy in the liver. Upon fasting, BNIP3 stimulates LC3B translocation from the nucleus to the cytosol for mitochondrial autophagosome degradation (Springer et al., 2021). FUNDC1 is a mitophagy receptor present in the outer membrane of the mitochondria (Bi et al., 2024), and FUNDC1 deficiency in hepatocytes reduces mtDNA stability and promotes cascade activation and inflammation (Zhou et al., 2019).

#### Hepatic endoplasmic reticulum (ER)–mitochondrial interactions

The ER and mitochondria interact at contact sites known as mitochondria-associated membranes or mitochondria–ER contacts, where they exchange phospholipids and calcium, thus modulating key signaling pathways and regulating cellular homeostasis (Filadi et al., 2017). Reduced interactions and calcium exchange between the ER and the mitochondria represent early events in the liver of mice with diet-induced obesity. Disruption of the communication between the ER and the mitochondria triggers hepatic insulin resistance and steatosis. Conversely, enhancing the interactions between the ER and the mitochondria can prevent diet-induced glucose intolerance (Beaulant et al., 2022).

#### Phospholipid of mitochondria

Similar as the plasma membrane, the mitochondrial membrane is mainly composed of three classes of lipids: glycerophospholipids containing a glycerol backbone, sphingolipids containing a sphingosine backbone, and sterols containing a four-ringed structure. Mitochondria could synthesize several lipids such as phosphatidylglycerol (PG), cardiolipin and in part phosphatidylethanolamine (PE), phosphatidic acid and CDP-diacylglycerol on their own. But other mitochondrial membrane lipids such as phosphatidylcholine (PC), phosphatidylserine (PS), phosphatidylinositol, sterols and sphingolipids have to be imported from cytosol. These phospholipids are crucial for maintaining membrane electrical potential, mitochondrial homeostasis and function (van der Veen et al., 2017).

PE and PC are the main two types of phospholipids within the mitochondrial membrane. PE is highly enriched and accounts form 40% to total phospholipids. Approximately, 70% of PE resides in the outer side of the OMM (van der Veen et al., 2017). PC is synthesized from the CDP-choline pathway and could be obtained by conversion PE to PC via phosphatidylethanolamine N-methyltransferase (PEMT). In PEMT deficient mice, PC/PE ratio is decreased. Mitochondria are smaller and more elongated, accompanying increased respiration and cytochrome oxidase and succinate reductase production in liver (Li et al., 2023). When challenged with high-fat diet, PEMT deficient mice could develop more severe NAFLD than their littermates (Li et al., 2023). Apart from that, PC could be also delivered to mitochondria via Stad7. *In vitro*, knockdown of Star7 reduces PC content in mitochondria whereas overexpression of Stad7 increases PC content and induces mitochondrial fusions (Rojas et al., 2021).

PS is synthesized in mitochondria via phosphatidylserine decarboxylase (PSD) or translocated from ER (Johnson et al., 2023). In PSD deficient mice, mitochondrial mass is small and fragmented and mice die in early stage of embryonic development (Steenbergen et al., 2005). With regard to transfer, Mfn2 is the mediator that specifically extracts PS from ER to mitochondrial membrane. In case of Mfn2 deficiency, PS transfer is abrogated, triggering ER stress and development of NASH (Hernández-Alvarez et al., 2019).

PG is synthesized in the mitochondria and transported to ER for maturation. PG is required for cardiolipin synthesis to maintain cristae structure. Lysophosphatidylglycerol Acyltransferase 1 **(**LPGAT1), PG an acyltransferase that catalyzes located in ER coordinates transport of PG to mitochondrial (Sun et al., 2023a). In LPGAT^−/−^ mice, LPGAT1 ablation disrupted the transportation of matured PG back to mitochondria is disrupted, resulting in loss of crista structure and respiratory function (Sun et al., 2023a).

#### The mitochondria–lysosome-related organelle

Very recently, a novel organelle was identified in dedifferentiated primary mouse hepatocytes cultured *in vitro*. When primary mouse hepatocytes were cultivated for up to 7 days, the mitochondria underwent dynamic remodeling, exhibiting increased elongation and fragmentation. An organelle with a hybrid “mitochondria–lysosome-like structure” was identified, named the mitochondria–lysosome-related organelle. This organelle is distinct from the mitophagosome and functions in mitochondrial degradation during cell dedifferentiation (Ma et al., 2023).

#### Mitochondrial transfer

Very interestingly, mitochondria could be transferred among cells via tunnelling nanotubes. Alternatively, mitochondria can be transferred in packaged vesicles and released to the extracellular compartment. The released vesicles are captured by recipient cells and degraded by the lysosome. Thus, the content of mitochondria is captured by recipient cells (Borcherding and Brestoff, 2023). Although the biological meaning of mitochondria transfer is not understood, to some extent, it acts as a “gain of function” mode for recipient cells in the aspect of exogenous respiration and rescue of inherited mitochondria defect. For instance, *in vitro*, the addition of purified mitochondria to NDUF4-deficient mice could restore mitochondria respiration in peritoneal macrophages (Borcherding et al., 2022). Furthermore, massages delivered via mitochondria transfer could modulate the fate of recipient cells. For example, mitochondria are transferred from osteoblasts to their progenitors could promote its differentiation to osteoblasts for bone formation (Mohammadalipour et al., 2020).

**FIGURE 2:**
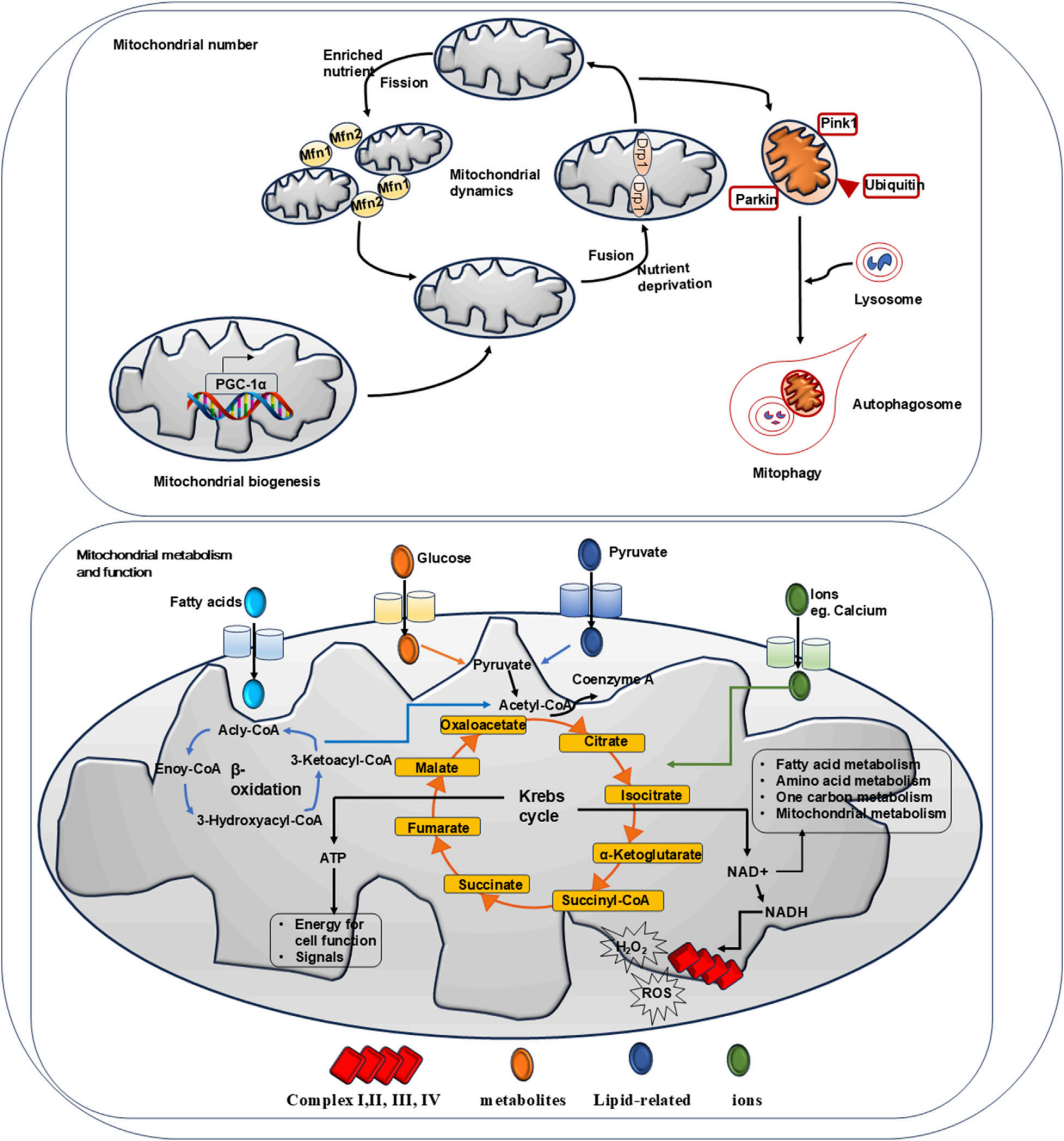
Summary of mechanisms for mitochondrial homeostasis. Mitochondrial biogenesis, mitochondrial dynamics and mitophagy govern the number of mitochondria. Physically, AMPK activation promotes PGC-1α activation which induces target gene expression to facilitate mitochondrial biogenesis. When exposed to nutrient-rich environmental conditions, mitochondrial fission factor 1 and 2 (Mfn-1 and Mfn-2) regulate fission to reduce bioenergetic efficiency and augment mitochondrial uncoupling. Under nutrient deprivation, dynaminrelated proteins 1 (Drp-1) modulate mitochondrial fussion to protect its function. Via phosphorylated PINK and PARKIN, mitochondrial mitophagy controls mitochondrial turnover and recycling, ensuring hepatic homeostasis by eliminating long-lived or damaged mitochondria. Physically, mitochondrial is the key organelle to produce ATP via Krebs cycle and β-oxidation. Metabolites such like glucose and pyruvate are fluxed into mitochondrial via transporters to participate Krebs cycle reaction. Ions such like calcium are entered via VDAC channel to mediate bio-chemical reactions. Fatty acids are entered to carry out β-oxidation. Via Krebs cycles and β-oxidation, ATP is produced to support cell function. NAD + generated from chemical reaction participates in cell metabolism. NADH passes through respiratory complex chain to produce ROS and H_2_O_2_.

Figure 2 summarizes a series of mechanisms to maintain mitochondrial homeostasis.

## Hepatic mitochondrial dysfunction in NAFLD

Mitochondrial homeostasis is disrupted in the early stage of NAFLD and becomes dysfunctional along with the transition from NAFL and non-alcohol steatohepatitis (NASH) to more severe NAFLD, accompanying with significant morphological changes, impaired biogenesis and dynamics, increased oxidative stress, and inflammation.

### Mitochondrial adaptation

Using high-resolution respirometry to measure oxygen flux, mitochondrial function was studied in the whole liver tissue and *ex vivo* isolated liver mitochondria from lean individuals and obese patients with different stages of NAFLD. When steatosis-resistant A/J mice were fed a high-fat diet (HFD) for 2, 6, and 30 days, respectively, 13 OXPHOS genes were increased shortly after 2 days of the HFD, as evidenced by complementary DNA microarray. Accompanying that, hepatic ATP content was increased, suggesting elevated mitochondrial function (Poussin et al., 2011). In line with that, the uncoupled respiration to β-oxidation and TCA cycle activity was 85% higher in individuals with obesity compared with lean controls. Likewise, OXPHOS activity was increased despite low intrahepatic triglyceride content, indicating mitochondrial adaptation to elevated lipid exposure in case of obesity (Koliaki et al., 2015). In another study, liver biopsies were obtained from patients with obesity undergoing bariatric surgery and lean controls. Hepatic mitochondrial respiratory capacity by high-resolution respirometry and mtDNA/nDNA content by quantitative polymerase chain reaction were much higher in patients with obesity than in controls (Pedersen et al., 2022). Taken together, these data imply that the mitochondria undergo adaptation or plasticity in order to reduce hepatic fat accumulation in the early stage of NAFLD.

### Defective mitophagy

Mitophagy governs mitochondrial quality via selective clearance of damaged or excess mitochondria. A growing body of evidence has revealed the impaired mitophagy and change in mitochondrial function in the transition from NAFL to NASH (Koliaki et al., 2015). Using high-resolution respirometry, mitochondrial respiration was measured in liver biopsies from patients with obesity without NAFL or with NAFL or NASH. The maximal mitochondrial respiratory rate was comparable between patients with obesity with or without NAFL. However, mitochondrial mass was greater, but maximal respiration was 31%–40% lower, in patients with NASH than in patients with NAFLD (Koliaki et al., 2015). Changes in mitochondrial structure, reduced ATP production, and increased oxidative stress become more pronounced in the liver following disease progression toward NASH (Ozer et al., 1999; Koliaki et al., 2015).

Physically, upon cellular entry, fatty acids are recruited to the mitochondria for β-oxidation or esterified to triglycerides in the cytosol. In the context of excess nutrients, triglyceride accumulation and reduced fatty acid oxidation are the hallmarks of NAFLD. Acyl-coenzyme A-dependent lysocardiolipin acyltransferanse 1 (ALCAT1) catalyzes the remodeling of cardiolipin, which is a mitochondrial phospholipid involved in membrane biogenesis and facilitates the catalyzation of fatty acid β-oxidation by mitochondrial trifunctional protein. Western blot has demonstrated elevated hepatic ALCAT1 expression in NAFLD models, including HFD-fed wild-type mice and diabetic db/db mice. Ectopic ALCAT1 overexpression in hepatocytes caused cardiolipin peroxidation, resulting in mitochondrial dysfunction and steatosis development. By contrast, ALCAT1 deficiency promoted mitophagosome biogenesis via upregulation of PINK, preserved mitochondrial structure, and attenuated steatosis (Wang et al., 2015).

As described above, Parkin is an essential regulator of mitophagy. In experimental models of NAFLD induced by a high-fat/high-calorie diet, an initial increase in PINK1/Parkin-mediated mitophagy was observed as a protective response to combat hepatic lipid buildup. However, prolonged HFD exposure resulted in a decline in PINK1/Parkin expression, compromising the efficiency of mitophagy and exacerbating liver damage, ultimately progressing to NASH (Li et al., 2024). In wild-type mice fed an HFD and palmitate acid-treated hepatocytes, macrophage-stimulating 1 expression was upregulated, which inhibited Parkin expression and reduced mitophagy (Zhou et al., 2019). Conversely, Mst1 knockdown restored mitophagy function, reducing liver injury and enhancing hepatocyte viability (Zhou et al., 2019).

A recent study revealed the mortality factor 4-like protein 1 (MORF4L1, also called MRG15) as a novel regulator of mitophagy. MRG15 is a component of the NuA4 histone acetyltransferase complex, which is involved in transcriptional activation of certain genes, principally by acetylation of the nucleosomal histones H4 and H2A. MRG15 resides in OMM and its expression is elevated in the liver of patients with NASH (Tian et al., 2022). In a murine model of NASH, the increased MRG15 protein interacted with and deacetylated mitochondrial Tu translation elongation factor (TUFM) that activated the ClpXP protease system for protein degradation. By contrast, MRG15 blockade stabilized TUFM-sustained mitophagy and attenuated NASH progression (Tian et al., 2022).

DEAD-box protein 5 (DDX5) is an ATP-dependent RNA helicase that interacts with E2F1 to stimulate the transcription of Atg4B to modulate mitophagy (Zhang et al., 2023). Nevertheless, one study showed that DDX5 expression was downregulated in diet-induced NASH models and palmitic acid-exposed hepatocytes (Zhang et al., 2023). In another study, delivery of mesenchymal stem cell-derived extracellular vesicles with enriched DDX5 promoted mitophagy, mitochondrial function, and hepatocyte proliferation in aged mice undergoing partial hepatectomy (Zhang et al., 2023). Therefore, the roles of DDX5 in mitophagy are to be covered.

### ROS and oxidative stress

ROS are byproducts of OXPHOS and encompass a range of compounds, including superoxide anion radical (O2•−), hydrogen peroxide (H_2_O_2_), and hydroxyl radical (HO•), as well as various peroxides, such as those found in nucleic acids, lipids, and proteins (Cadenas and Sies, 2023). In NAFLD, ROS elevation leads to prolonged mitochondrial permeability transition pores (mPTP) opening, causing dissipation of the mitochondrial membrane potential (ΔΨm). This results in an influx of water and ions into the mitochondrial matrix, leading to mitochondrial swelling and eventual outer membrane rupture. Subsequently, a rapid increase in ROS production during the “burst phase” ensues, causing oxidative damage to mitochondrial DNA, proteins, and lipids. In addition, ROS triggers mitophagy by prompting Parkin to move from the cytoplasm to the impaired mitochondria, activating the PINK1/Parkin pathway (Lin et al., 2019).

### Imbalanced fusion and fission

Of note, when hepatocytes are exposed to high levels of cellular stress with excessive nutrients and free fatty acids, the mitochondrial network becomes more disintegrated through increased fission (Liesa and Shirihai, 2013). An *in vitro* study showed that exposure of hepatocytes to saturated palmitate resulted in triglyceride accumulation, mitochondrial fragmentation, loss of transmembrane potential, cytochrome-c release into the cytoplasm, and increased ROS activity (Eynaudi et al., 2021).

Similar to these *in vitro* findings, enhanced fission machinery and decreased mitochondrial respiratory capacity have been observed in the liver of db/db mice and rats fed an HFD (Galloway et al., 2014; Piacentini et al., 2018). In transgenic mice expressing the dominant-negative fission mutant DLP1-K38A in a doxycycline-inducible manner, DLP1-K38A was induced by an HFD. Galloway et al. (2014) found that inhibiting mitochondrial fission protected against liver steatosis, alleviated HFD-induced oxidative stress, and reduced hepatic damage. Moreover, Takeichi et al. (2021) demonstrated that hepatic deletion of mitochondrial fission factor-induced ER stress impaired triglyceride secretion in the liver, both *in vivo* and *in vitro*. They further showed that mice lacking mitochondrial fission factor in hepatocytes exhibited increased susceptibility to NASH induced by the HFD, primarily due to enhanced hepatic apoptosis resulting from ER stress and reduced triglyceride excretion from hepatocytes (Takeichi et al., 2021). These findings provide novel insights into the role of mitochondrial fission in NASH development.

In summary, the progression from NASH to more severe forms of NAFLD is characterized by a cascade of mitochondrial changes, including impaired function, altered biogenesis and dynamics, and mitochondrial-induced apoptosis and inflammation. Understanding these mitochondrial alterations is crucial for elucidating the pathophysiology of NAFLD progression and for developing targeted therapeutic strategies to mitigate liver damage in patients with advanced disease.

### Impaired cross-talk

When mitochondria undergo dysfunction, the communication to other organelles is also affected. During mitochondria fusion, binding of Mfn-2 to phosphatidylserine helps transfer of phosphatidylserine to mitochondria for phosphatidylethanolamine synthesis. In liver biopsy of NASH patients, Mfn-2 expression was reduced. In mice with hepatic ablation of Mfn-2, phosphatidylserine transfer is abrogated, leading to ER stress, triglyceride accumulation and fibrosis (Hernández-Alvarez et al., 2019). In Cadmium-induced NAFLD mice, exposure of cadmium to hepatocytes damaged nanotube system, mitochondria-kinesin interaction and blocked mitochondrial transfer, all of which resulted in lipid accumulation in liver (Sun et al., 2023b).

**FIGURE 3:**
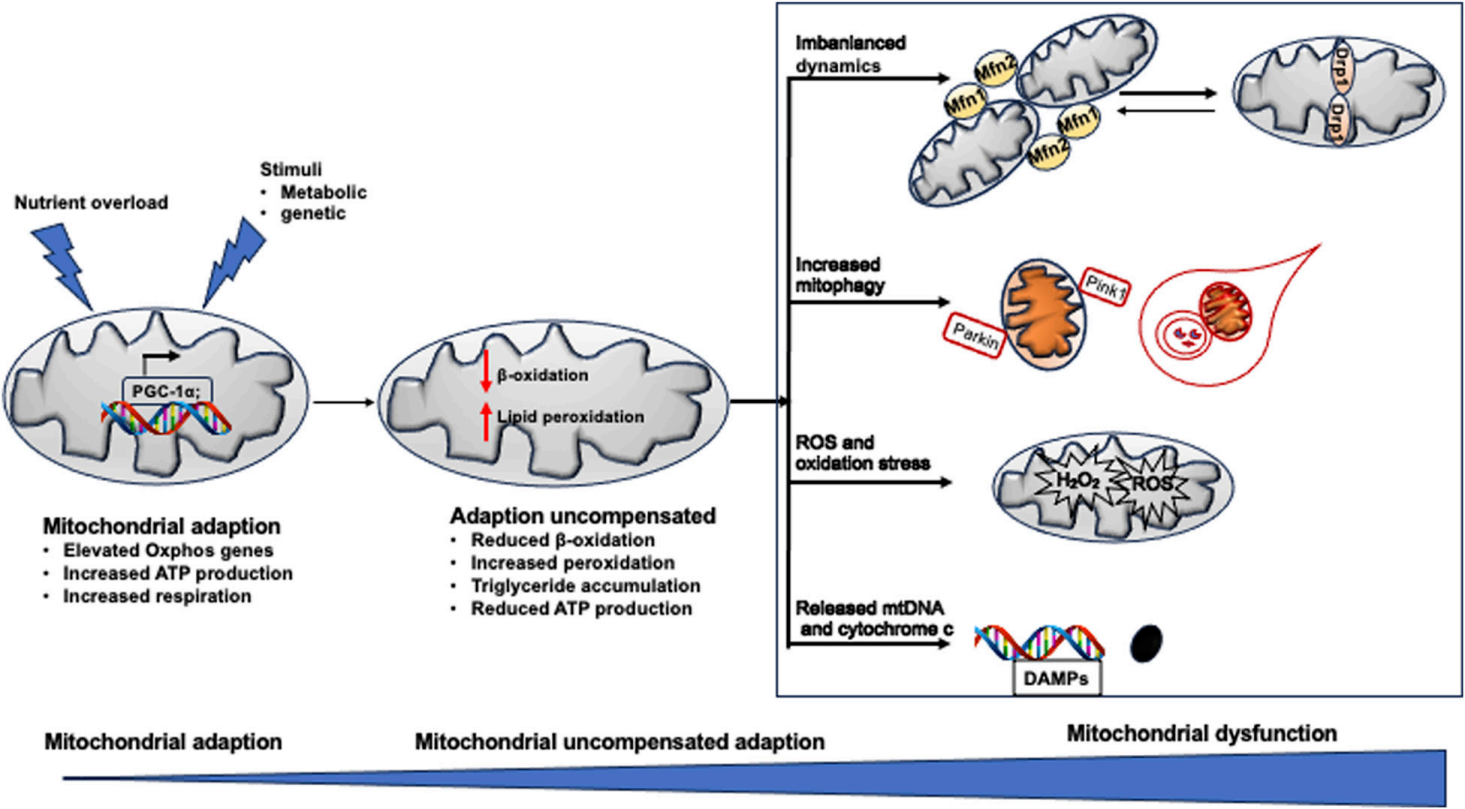
Mitochondrial dysfunction in NAFLD. When exposed to excess nutrient or stimuli, Oxphos gene expression is increased to adapt the metabolic change. When adaption could not compensate the pathological change, peroxidation become dominant in mitochondrial rather than β-oxidation, resulting in pronounced production of ROS, breakdown of mitochondrial fission and fusion, defected mitophagy. All of which induce mitochondrial damage and release mtDNA fragment (DAMPs) and cytochrome C. The death signals propagate hepatocyte injury and triglyceride accumulation in the liver, leading to NAFLD.

Figure 3 summarizes the dysfunctional mitochondria in the pathogenesis of NAFLD.

## Drugs targeting mitochondrial function in NAFLD

For most patients with NAFLD, the primary goal is to improve quality of life and prolong life expectancy. The second goal is to reduce hepatic fat deposition and inhibit the progression of inflammation and fibrosis. Pharmacological agents can be classified into five categories based on their mechanisms of action, namely lipid-lowering, glucose-lowering, anti-apoptotic, anti-inflammatory, and anti-fibrotic agents. Herein, we describe the clinical results of the most promising drugs and agents for the treatment of NASH and NAFLD, focusing on their effect on mitochondrial homeostasis.

### Pemafibrate

Pemafibrate is a selective peroxisome proliferator-activated receptor PPAR-α agonist. Upon ligand binding, hepatic PPAR-α induces mitochondrial fatty acid uptake and β-oxidation (Matsumoto et al., 2023). In a phase 2 randomized controlled trial, 118 patients with NAFLD were assigned to 0.2 mg pemafibrate or placebo for 72 weeks. Magnetic resonance elastography revealed that the liver fat content was decreased by 5.7% at week 48 and by 6.2% at week 72 in the pemafibrate group compared with placebo (Nakajima et al., 2021). Along with reduced fatty liver, alanine transaminase and low-density lipoprotein cholesterol were significantly lower in the treatment group (Nakajima et al., 2021).

### Mitochondrial pyruvate carrier (MPC) inhibitors

Thiazolidinediones (TZDs) are a class of anti-diabetic drugs. It activates PPAR-γ transcription and its subsequent increased glucose transporter (GLUT4) expression to enhance glucose uptake in hepatocytes and adipocytes (Colca et al., 2023). Interestingly, TZDs have been found to specifically inhibit the activity of mitochondrial pyruvate carrier (MPC) (Divakaruni et al., 2013).

Pyruvate is the key metabolite linking glycolysis and oxidative phosphorylation in hepatocytes. The MPC components, MPC1 and MPC2, are discovered to be located in the mitochondrial and function in transporting pyruvate produced in glycolysis into mitochondrial matrix (Tavoulari et al., 2023). In NAFLD patients, a positive correlation between MPC1 levels and hepatic lipid content was observed (Gao et al., 2023). From mechanistic insight, inhibition of MPC1 ammolites protein function by introducing lactylation modification onto targeted proteins such like fatty acid synthase. Ultimately, suppression of MPC1 results in reduced lipid accumulation and inflammation in hepatocytes. Based on that, insulin sensitizer MDSC-0602K is specifically designed to preferentially target MPC while minimizing direct binding to the transcriptional factor PPARγ. In a multicenter, double-blinded RCT, 392 biopsy-confirmed NASH and fibrosis (F1-F3) patients were recruited and administered different doses of MSDC-0602K (0–250 mg daily). After 52 weeks of treatment, MSDC-0602K significantly improved hyperglycemia, and reduced markers of liver injury. Nonetheless, NAS was not changed compared with placebo (Harrison et al., 2020).

#### Glucagon-like peptide-1 (GLP-1) receptor agonists

GLP-1 is an incretin secretory molecule produced by enteroendocrine L cells. Acting through the GLP-1 receptor, the physical roles of GLP-1 and its agonists are multifaceted, including the promotion of glucose-induced insulin secretion, retarding gastric motility, protecting endothelial cell function, and inhibiting inflammation (Drucker, 2018; Laurindo et al., 2022). Therefore, GLP-1 receptor agonists have been widely applied in the treatment of type 2 diabetes mellitus. Administration of liraglutide or semaglutide to mice with diet-induced steatosis improved the NASH activity score along with reducing fat deposition and inflammation in the liver (Niu et al., 2022; Newsome and Ambery, 2023).

For mechanistic insight, liraglutide to mice upregulated the expression of PGC-1α (peroxisome proliferator-activated receptor-γ coactivator-1α), which is a potent transcription factor for mitochondrial biogenesis and function (Wu et al., 2022). In parallel, in a murine model of NASH induced by a high-fructose and high-trans-fat diet, exenatide treatment decreased TCA cycle flux and improved lipid metabolism (Kalavalapalli et al., 2019). The addition of liraglutide partially rescued mitochondrial dysfunction by reducing ROS, inhibiting NLRP3 activation, and augmenting mitophagy in HepG2 cells treated with palmitic acid (Yu et al., 2019).

In patients with type 2 diabetes mellitus with co-existing NAFLD, administration of liraglutide (Feng et al., 2017; Yan et al., 2019; Guo et al., 2020) or dulaglutide (Kuchay et al., 2020) resulted in significant fat content in the liver compared with placebo (Kuchay et al., 2020), insulin glargine (Yan et al., 2019; Guo et al., 2020), or gliclazide (Feng et al., 2017). In overweight patients with NAFLD, treatment with liraglutide (Armstrong et al., 2016; Khoo et al., 2017) not only reduced fat content (Khoo et al., 2017), but it also improved fibrosis resolution in the liver (Armstrong et al., 2016). In patients with biopsy-confirmed NASH and liver fibrosis of stage 1, 2, or 3 (Newsome et al., 2021; Loomba et al., 2023) or advanced steatosis (Flint et al., 2021), subcutaneous injection of semaglutide failed to improve fibrosis. Recently, a phase 2a randomized controlled trial was conducted to compare the therapeutic effects of efinopegdutide (GLP-1 and glucagon receptor co-agonist) and liraglutide. After 24 weeks of treatment in patients with NAFLD, efinopegdutide was more powerful than semaglutide in reducing liver fat content, as assessed by magnetic resonance imaging-estimated proton density fat fraction (Romero-Gómez et al., 2023). Whether efinopegdutide attenuates fibrosis progression remains to be explored in the future.

#### Cotadutide

Cotadutide is the GLP-1R/Glucagon receptor (GcgR) dual agonist (Boland et al., 2020). Besides its beneficial effects via GLP-1 signaling, Gcg signaling improved mitochondrial turnover, glucose and lipogenic metabolism and function in mice (Boland et al., 2020). In line with *in vivo* results, a phase 2 study was performed to assess the therapeutic efficacy of cotadutide in NAFLD. Overall, 834 obese and T2DM patients were randomized with placebo or different dosages of cotadutide. After 54 weeks of treatment, cotadutide treatment decreased HbA1c and fibrosis-4 index compared with placebo (Nahra et al., 2022).

Table 1 summarizes of the results of randomized clinical trials of drugs in the treatment of NAFLD.

**TABLE 1:** Summary of the results of randomized clinical trials of drugs in the treatment of NAFLD.

Drug name	Type of clinical study	Features of patients	Number of patients	Duration of treatment	Main findings
PPARα agonist					
Pemafibrate (Nakajima et al., 2021)	Double-blind, RCT	NAFLD	118	72 weeks	Pemafibrate reduced liver stiffness quantified by MRI but did not decrease liver fat content
Mitochondrial pyruvate carrier inhibitor					
MDSC-0602K (31697972)	Double-blind RCT	NASH, F1-F3	392	52 weeks	MSDC-0602K improved hyperglycemia and liver injury index, but did not demonstrate statistically significant effects on primary and secondary liver histology endpoints
GLP-1 receptor agonists					
Liraglutide (Armstrong et al., 2016)	Double-blind RCT	Overweight with clinical evidence of NAFLD	52	48 weeks	Improved histological resolution; less progression to fibrosis (9% vs 36%)
Liraglutide, Gliclazide (Feng et al., 2017)	Open-label, prospective, randomized	T2DM with NAFLD	87	24 weeks	Liraglutide resulted in greater hepatic fat content reduction than gliclazide
Liraglutide (Khoo et al., 2017)	Randomized to a supervised diet programing	Obese with NAFLD	24	26 weeks	Liraglutide was effective as lifestyle modification in the aspect of reduction of liver fat content
Liraglutide, Sitagliptin, Insulin-glargine (Yan et al., 2019)	Randomized, active-controlled, parallel-group, open-label	T2DM with NAFLD	72	26 weeks	Both liraglutide and sitagliptin, but not insulin glargine, reduced body weight, hepatic fat content, and adiposity in visceral adipose tissue
Dulaglutide (Kuchay et al., 2020)	Randomized, open-label, parallel-group	T2DM with NAFLD	64	24 weeks	Dulaglutide significantly reduces liver fat content but no significant reduction in liver stiffness, serum AST and serum ALT levels
Liraglutide, Insulin glargine (Guo et al., 2020)	Prospective, RCT	T2DM with NAFLD	96	26 weeks	Compared with placebo, Liraglutide treatment reduced hepatic fat content while insuline glargine did not
Semaglutide (Newsome et al., 2021)	Double-blind, RCT	Biopsy-confirmed NASH and liver fibrosis of stage F1, F2, or F3	320	72 weeks	Semaglutide resulted in a significantly higher percentage of patients with NASH resolution than placebo, but did not improve fibrosis stage compared with placebo
Semaglutide (Flint et al., 2021)	Double-blind, RCT	Liver stiffness 2.50–4.63 kPa by MRE and liver steatosis ≥10% by MRI	67	48 weeks	Semaglutide reduced hepatic fat content and steatosis but had not changed liver stiffness, compared with placebo
Semaglutide (Loomba et al., 2023)	Double-blind, RCT	Biopsy-confirmed NASH-related cirrhosis and BMI ≥27 kg/m^2^	71	48 weeks	Semaglutide did not significantly improve fibrosis or achievement of NASH resolution compared with placebo
Efinopegdutide, Semaglutide; (Romero-Gómez et al., 2023)	Randomized, active-comparator-controlled, parallel-group, open-label	NAFLD	145	24 weeks	Treatment with efinopegdutide 10 mg weekly led to a significantly greater reduction in LFC than semaglutide 1 mg weekly
GLP-1R/GcgR agonist					
Cotadutide (36,228,195)	RCT	Obese, T2DM	834	54 weeks	Cotadutide significantly decreased HbA_1c_ and body weight, improved lipid profile, AST and ALT levels, propeptide of type III collagen level, fibrosis-4 index, and nonalcoholic fatty liver disease fibrosis score

MRI, magnetic resonance imaging; RCT, randomized and placebo-controlled clinical trial; NAFLD, non-alcoholic fatty liver disease; NASH, non-alcohol steatohepatitis (NASH).

## Perspective

NAFLD is a complex disorder that involves metabolic dysregulation and inflammatory and fibrotic pathways. Although substantial progression has been made in the treatment of this disease, more effective treatments for NASH are still needed. Moreover, side effects are still common with the available pharmaceuticals. Mitochondrial homeostasis governs liver physiology and function. How to balance its phospholipid composition in membrane and how to keep physical metabolism and function under control are crucial for mitochondria homeostasis. Mitochondria transplantation is considered as a therapeutic approach in treating NAFLD is under investigation. However, how to isolate functional mitochondria is not solved yet. Moreover, further preclinical and clinical research on the maintenance of mitochondrial homeostasis is absolutely required for the treatment of NAFLD and NASH.
